# Pan-cancer analysis of FBXW family with potential implications in prognosis and immune infiltration

**DOI:** 10.3389/fimmu.2022.1084339

**Published:** 2022-12-14

**Authors:** Tingting Huang, XIaoxiao OuYang, Jiwei Li, Bingbing Shi, Zhengda Shan, Zhiyuan Shi, Zhangru Yang

**Affiliations:** ^1^ School of Medicine, Xiamen University, Xiamen, China; ^2^ Central Laboratory, Clinical Medical College & Affiliated Hospital of Chengdu University, Chengdu University, Chengdu, China; ^3^ Department of Critical Care Medicine, The Affiliated Hospital of Putian University, Putian, China; ^4^ School of Medicine, Sun Yat-Sen University, Shenzhen, China; ^5^ Department of Radiation Oncology, Shanghai Chest Hospital, Shanghai Jiao Tong University, Shanghai, China

**Keywords:** FBXW family, pan-cancer, prognosis, immune infiltration, therapeutic target

## Abstract

**Background:**

The F-box and WD repeat domain containing (FBXW) family of SCF E3 complexes has 10 members that are responsible for ubiquitination and degradation of substrate proteins involved in cell cycle regulation and tumorigenesis. Among them, FBXW1 (also called b-TrCP1/BTRC) and FBXW7 are the central proteins in this category. However, there is still a lack of elaborate exploration of the contribution of FBXW family members, especially FBXW1 and FBXW7, in various tumor types.

**Methods:**

In this present study, we preliminarily analyzed the genetic structure characteristics of the FBXW family, and systematically investigated their expression patterns and clinical correlations based on the TCGA pan-cancer data. Survival analysis of FBXWs was also conducted through the Kaplan-Meier method. In addition, we assessed their immune infiltration level through immune-related algorithms like Timer and xCell.

**Results:**

There were obvious genetic heterogeneity and different clinical traits in FBXW family members. Moreover, we found that FBXW family genes may be useful in predicting prognosis and therapeutic efficacy using survival analysis. In addition, the immune infiltration of FBXW family was also clearly illustrated in this study. The results showed these genes were closely involved in immune components such as immune score, immune subtypes, tumor-infiltrating lymphocytes and immune checkpoints. Notedly, FBXW1 as an oncogene and FBXW7 as a tumor suppressor gene also show opposite relationships on immune cells.

**Conclusion:**

Our results provided valuable strategies to guide the therapeutic orientation concerning the role of FBXW family genes in cancer.

## Introduction

Cancer is a crucial cause of death and a great barrier to improving life expectancy and quality (1, 2). Based on the predictions of Global Cancer Statistics 2020, there are almost 19.3 million new cancer illness and 10.0 million carcinoma-related deaths (3). In addition, the incidence and mortality of cancer are growing dramatically. Ubiquitin Proteasome System (UPS), which can promote protein binding to ubiquitin (Ub) and transfer to proteasome for proteolysis, has been involved in the progression and development of cancers as the UPS controls the degradation of numerous tumor suppressors and oncogenes (4, 5).UPS with highly conserved protein degradation mechanisms plays critical roles in various physiological processes, including signal transduction, cell cycle, DNA replication, transcriptional regulation, as well as cell death and immune response (6). Dysregulation of this system has been implicated in several human malignant transformation (7). The process of degradation is carried out in three strides by multiple components which includes Ub activating E1 enzyme, Ub conjugating E2 enzyme, and Ub E3 ligase (4, 8).

Multitude of E3 ligases have been broadly involved in the cancer-related processes, due to the characteristic of their specific substrate recognition (9). A representative example of E3 enzyme is a conserved ubiquitin ligase complex known as SCF E3 ligases which is composed of three invariable elements- SKP1, Cullin, RBX1 and a variable component -FBP (10). The 69 FBPs family members can be classified as three subclasses as follows: FBXW, FBXL, and FBXO. The FBXWs containing WD40 repeats play a pivotal role in targeting proteins associated with cell cycle regulation and tumorigenesis (11). The FBXW family has ten members: FBXW1(also known as β-TrCP1), FBXW2, FBXW4, FBXW5, FBXW7, FBXW8, FBXW9, FBXW10, FBXW11(also known as β-TrCP2), and FBXW12, in which β-TrCP and FBXW7 are central proteins (4, 12). FBXW1 and FBXW11 are two β-TrCP homologs with similar biological effects which mainly function as adaptors to recognize specific substrates such as β-catenin, CDC25A, IκB and DEPTOR (12, 13). β-TrCPs have been demonstrated to role as oncogene or tumor suppressor depending on the diversity of substrates. Susanne et al. discovered that overexpression of FBXW1 was vital mediator of constitutive NF-κB activation leading to chemoresistance in pancreatic cancer cells (14). In particular, FBXW1 targets Snail and EZH2 ubiquitination which are involved in epithelial-to-mesenchymal transition in cancers (15). FBXW7 roles as a tumor suppressor on account of promoting the degradation of various oncogenes including Cyclin E, c-Jun, Notch, mTOR, c-Myc, etc. (16). Zhan et al. studied that FBXW7 as a tumor suppressor negatively regulates ENO1-induced cell proliferation and migration in colorectal cancer cells (17). In addition, the high frequency mutation of FBXW7 was found in multiple cancers. For example, the rate of mutation is approximately 35% in cholangiocarcinomas and 30% in T-cell acute lymphocytic leukemia (18, 19). Additionally, FBXW1 and FBXW7 co-coordinate the apoptotic process of cells by ubiquitinating MCL1 (20). However, the full picture of FBXW family in pan-cancer, especially these central proteins, has yet to be explained.

In this study, we primarily investigated the structural of FBXW members and comprehensively analyzed the expression levels and prognostic value of FBXWs in pan-cancer *via* the Cancer Genome Atlas (TCGA) database. Furthermore, the relationship between FBXWs expression and tumor microenvironment (TME) were evaluated by TIMER database. Besides, we also explored the correlation between FBXWs expression levels and immune subtype, drug sensitivity in multiple cancers. FBXW1/7 as central proteins, their relationship with immune infiltration was also conducted in this research. Together, our study revealed the detailed expression patterns and overall picture of the immune infiltration of FBXW gene family in pan-cancer, which provide further insights for FBXWs as potential therapy targets in Pan-cancer.

## Materials and methods

### Analysis of the main characteristics of FBXW family members in *Homo sapien*

The phylogenetic tree of the FBXWs was constructed in this study using MEGA 7.0.26 with the neighbour-joining (NJ) method and 1000 bootstrap replications (21). Then, it was visualized by the online tool TBtools (22). GFF3 files (General Feature Format Version 3) were downloaded in the ensemble database for the structural analysis of FBXWs. The characteristic motifs of FBXWs were conducted by MEME (http://meme-suite.org/tools/meme) software. Furthermore, TBtools was also used for analyzing the gene structure of all FBXWs and SWISS-MODEL Interactive Workshop was employed for the prediction of the tertiary structure of FBXW proteins (23, 24).

### TCGA pan-cancer atlas data profile

We used the UCSC Xena (https://xenabrowser.net/) to analyse the TCGA pan-cancer data of FBXW family members, including gene expression, clinicopathological data, molecular subtype, survival data and stemness score (25). The perl software was used to integrate the FBXW family expression and the Wilcox test was also taken to assess the difference between normal and tumor tissues. “*”, “**”, “***”, indicate P-value <0.05, <0.01, <0.001, respectively. “Ggpubr” and “pheatmap” packages were used for illustrating the expression pattern of FBXWs with a form of box plot and heatmap. Correlation analysis among FBXW family genes was performed by R-package “corrplot”. Furthermore, “ggplot2” package was use for the examination the relationship between the clinical stages and grades and FBXW family, and the GSCA database was performed to analysis the correlation between FBXW family expression and the subtypes of various tumors.

### Survival and cox analysis of expression of FBXW family

The Kaplan-Meier method and the log-rank test were employed to evaluate the survival situation of FBXW members in various tumors. “survival” and “survminer” R package were used for evaluation the prognosis role of these genes. Besides, we also conducted a COX analysis to establish the association between FBXWs expression and prognosis of pan-cancer. Finally, the forest plot was drawn using “survival” and “forestplot” packages.

### Genetic alteration analysis

cBioPortal platform integrates data from comprehensive tumor genome studies, including TCGA and ICGC large tumor projects, with data from more than 28,000 samples (26). In this study, we used the cBioPortal database to explore the mutation frequency and form of FBXW family members.

### Tumor microenvironment analysis

“Estimate” and “limma” R packages were applied to investigate the immune score, estimate score, and stromal score of different tumor cases based on TCGA expression data. Spearman test was applied to demonstrate the relationship between FBXW family genes and these scores. Furthermore, an association analysis between FBXWs expression and RNA stemness score (RNAss), DNA stemness score (DNAss) was established using the Spearman’s method and “limma” package.

### Immune infiltration cells and immune checkpoints correlation analysis

Immuneeconv, which is able to integrate the two latest algorithms, TIMER and xCell for reliable immune score evaluation, was used in this study for evaluating the immune infiltration level of FBXWs in Pan-cancer. A spearman correlation analysis of the immune score or immune infiltration cells and FBXW family genes expression in various tumors was also conducted in this research. Furthermore, the associations of FBXW family genes with representative immune checkpoints selected were also generated using spearman correlation test. ∗P< 0.05, ∗∗P< 0.01, and ∗∗∗P< 0.001.

### Immune subtype and drug sensitivity correlation analysis of FBXW family genes

The “limma” “ggplot2” and “reshape2” R package were employed for the conduction of the immune subtype analysis of FBXW family genes. Additionally, CellMiner™ (https://discover.nci.nih.gov/cellminer/home.do) database was applied for the drug sensitivity analysis of FBXWs, and “impute”, “limma”, “ggplot2”, and “ggpubr” R packages were used for the data process and visualization.

### Cell culture

All cells used in the study were purchased from the American Type Tissue Collection (ATCC) and cultured according to the manufacturer’s instructions (27). Cell mediums were commercially obtained from Solarbio Science & Technology Co., Ltd (Beijing, China).

### qRT-PCR analysis

Total RNA extraction and cDNA reverse transcription were elaborated in previous studies (28). The above steps were taken by the SPARKscript IISYBR Green qRT-PCR Kit (Shandong Sparkjade Biotechnology Co., Ltd.) instructions. Syber Green (Yeasen Biotechnology Co., Ltd.) was used for the qRT-PCR process with a 10ul reaction system.

FBXW1 forward, 5′- TCTCGAAGGCCGCTTACT -3′,

FBXW1 reverse, 5′- ATACCTGGATGCCAAATCA-3′;

FBXW7 forward, 5′- ACAACGCACAGTGGAAGTA -3′,

FBXW7 reverse, 5′-AGTGGGACATACAGGTGGA-3′

The 2^−ΔΔCt^ method was applied to calculate the relative expression levels of targeted genes. Dunnett’s t test was employed for the comparison between the experimental group and the normal group in this study. Each experiment was conducted in triplicate.

## Results

### Gene structure and motif composition of FBXWs in *Homo sapiens*

The F-box and WD repeat domain containing (FBXW) family, a key component of SCF-type (Skp1-Cullin1-F-box) ubiquitin ligase, specifically recognize the substrates and perform ubiquitination degradation, thereby affecting cell growth, differentiation, apoptosis and tumorigenesis (29, 30). In this study, we firstly investigated characteristics of 10 members of the FBXW family in *Homo sapiens* using uniport and GeneCards database, and the results showed that most of them located in cell cytoplasm and cytosol. Besides, the acquired Go term by the “pathway” module of GeneCards tool illustrated FBXW family members are involved in different biological response processes. For example, FBXW1 and FBXW4 were related to Protein polyubiquitination and Wnt signaling pathway, whereas FBXW7 was mainly involved in Vasculogenesis, DNA repair, Sister chromatid cohesion and Protein ubiquitination (**Table 1**). Besides, we found that these FBXWs shared same F-box and WD repeat domain with close interactions, and the enrichment analysis results demonstrated that they were primarily involved in ubiquitination-mediated degradation pathway (**sFigure 1**).

**Table 1:** Characteristics of 10 members of the *FBXW family* in *Homo sapiens* using uniport and GeneCards database.

Gene name	Ensemble	Protein Length (aa)	Subcellularlocation	Qualified GO term	Representative Substrates	Oncogene	Tumor suppressor gene
FBXW1	ENSG00000166167	605	Cytoplasm, Nucleus	Protein polyubiquitination; Signal transduction; Protein dephosphorylation; Wnt signaling pathway	STAT1, p63, Pro-caspase 3 Eml1, REST, CDC25A, CDC25B, Snail, IκB	COAD (31, 32), Hepatoblastoma (33), Melanoma (34), PAAD (14),	GBM (35) mUC (36)
FBXW2	ENSG00000119402	454	Cytosol	Proteolysis; Protein ubiquitination; Protein modification process	SKP2, p65, β-catenin, MSX2, EGFR		NSCLC (37–39), BRCA (40), PRAD (41)
FBXW4	ENSG00000107829	412	Cytosol	Regulation of mesenchymal cell proliferation; Protein polyubiquitination; Wnt signaling pathway	None	AML (42)	
FBXW5	ENSG00000159069	566	Cytoplasm; Cytosol	Mitotic nuclear division; Centrosome duplication; Protein ubiquitination	SASS6, EPS8, TSC2, SEC23B kinesin-13, DLC1, TSC2	STAD (43, 44) NSCLC (45)	
FBXW7	ENSG00000109670	707	Nucleus; Cytoplasm; Chromosome; Nucleoplasm	Vasculogenesis; DNA repair; Sister chromatid cohesion; Protein ubiquitination	c-Myc, Aurora A, Cyclin E, c-Myb, Notch, c-Jun, Mcl-1, mTOR		COAD (17, 46), LIHC (47, 48), T-ALL (49), NSCLC (50), STAD (51–55), PAAD (56, 57), RCC (58, 59), NSCLC (60), PRAD (61)
FBXW8	ENSG00000174989	598	Cytoplasm; Cytosol	Golgi organization; Dendrite morphogenesis Cell population proliferation; Protein ubiquitination;	MAP4K1, β-TrCP1, cyclin D1, CDK4, HPK1	PAAD (62)	STAD (63), COAD (64),
FBXW9	ENSG00000132004	458	Cytosol	None	None		
FBXW10	ENSG00000171931	1052	Cytosol	None	SOS, CBX5, CBX1		
FBXW11	ENSG00000072803	542	Cytoplasm; Nucleus	Mitotic spindle orientation; Cell cycle; Protein polyubiquitination; Nuclear migration; Protein dephosphorylation	CTNNB1, NFKBIA, IFNAR1, CEP68, RCAN1, CDC25A, CYTH1	COAD (65), ALL (66), BRCA (67), CC (68)	Osteosarcoma (69), Chondrosarcoma (70), NSCLC (60) STAD (71, 72), PRAD (73)
FBXW12	ENSG00000164049	464	Cytosol	None	IL-22R		

BRCA, breast invasive carcinoma; COAD, colon adenocarcinoma; LIHC, liver hepatocellular carcinoma; PAAD, pancreatic adenocarcinoma; PRAD, prostate adenocarcinoma; STAD, stomach adenocarcinoma; GBM, glioblastoma; mUC, urothelium carcinoma; T-ALL, T-cell acute lymphoblastic leukemia; NSCLC, non-small cell lung cancer; RCC, renal cell carcinoma; CC, cervical cancer.

**Figure 1:**
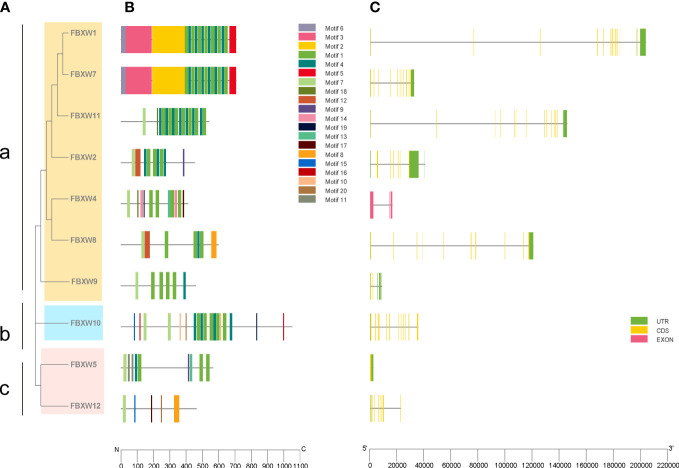
Phylogenetic relationships, conserved protein motifs and gene structures of the FBXW family members. **(A)** The phylogenetic tree was constructed based on the full-length sequences of *Homo sapiens* FBXW proteins. **(B)** Motifs distribution of the FBXW proteins. **(C)** Exon-intron structures of the FBXW genes. Green boxes indicate untranslated region; yellow boxes indicate coding sequences; pink boxes indicate exons and black lines indicate introns. a, b and c represent the three groups, respectively.

To further understand the phylogenetic relationship of human FBXW family, 10 amino acid sequences of FBXWs were used to construct phylogenetic tree in the research, and the gene family was divided into three groups. Group a was the largest members of the FBXW family, however, group b has only one member FBXW10 (**Figure 1A**). To explore the function of FBXWs, the conserved motifs of these 10 proteins were illustrated. The results showed 20 conserved motifs were detected in the FBXW family members (**Figure 1B**). Notedly, FBXW1 and FBXW7 had the same Motifs 2, 3, 5 and 6, suggesting that they may have similar functions in some biological processes. Furthermore, motifs 14 was discovered only in group a, and group b had the largest number of motifs. The reason of functional differences in the FBXWs of *Homo sapiens* was probably the clade-specific distribution of conserved motifs. Furthermore, the exon-intron patterns and tertiary structures of the 10 FBXWs were also explored in this study, suggesting the FBXW family members are structurally distinct (**Figure 1C**; **sFigure 2**).

### The expression levels of FBXW family in pan-cancer

**Figure 2:**
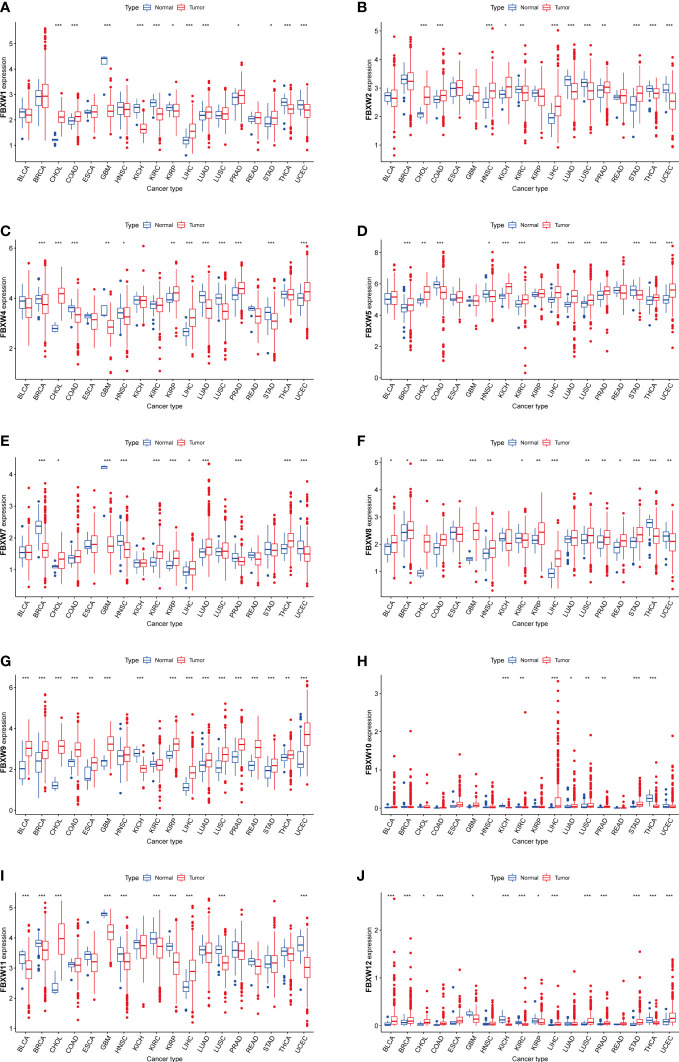
Boxplot of FBXW family **(A–J)** differential expression between cancer and adjacent normal tissues. The blue boxplots indicate the normal tissues. The red boxplots indicate the cancer tissues. ∗P< 0.05, ∗∗P< 0.01, and ∗∗∗P< 0.001.

We estimated the FBXWs differential expressions in Pan-cancer with RNA sequencing data of TCGA database. Our results indicated that FBXW1 was more expressed in colon adenocarcinoma (COAD), cholangiocarcinoma (CHOL), lung adenocarcinoma (LUAD), liver hepatocellular carcinoma (LIHC), Prostate adenocarcinoma (PRAD), and stomach adenocarcinoma (STAD). In contrast, a lower expression was found in kidney renal papillary cell carcinoma (KIRP), kidney chromophobe (KICH), kidney renal clear cell carcinoma (KIRC), glioblastoma multiforme (GBM), thyroid carcinoma (THCA) and uterine corpus endometrial carcinoma (UCEC) (**Figure 2A**). FBXW2 was more expressed in various cancers including CHOL, COAD, neck squamous cell carcinoma (HNSC), KICH, LIHC, PRAD, and STAD (**Figure 2B**).Meanwhile, lower expression was found in KIRC, LUAD, Lung squamous cell carcinoma (LUSC), THCA, and UCEC. The higher expression of FBXW4 was discovered in CHOL, KIRP, LIHC, PRAD, and UCEC. However, the lower level was in breast invasive carcinoma (BRCA), COAD, GBM, LUAD, LUSC, HNSC and STAD (**Figure 2C**). Additionally, FBXW5 was more expressed in BRCA, CHOL, LIHC, LUAD, LUSC, KICH, KIRC, PRAD, THCA and UCEC. The lower expression was in COAD, HNSC and STAD (**Figure 2D**). FBXW7 was higher expressed in CHOL, KIRC, KIRP, LIHC, LUAD, and THCA. Nevertheless, FBXW7 was lower expressed in BRCA, GBM, HNSC, PRAD, and UCEC (**Figure 2E**). We also analyzed that the higher expression level of FBXW8 was in several cancers, containing Bladder Urothelial Carcinoma (BLCA), BRCA, GBM, CHOL, COAD, HNSC, KIRP, LIHC, LUSC, STAD, PRAD and READ. However, the lower expression of FBXW8 was found was in LUSC, PRAD, READ, and STAD (**Figure 2F**). Besides, a higher FBXW9 expression was analyzed in BLCA, BRCA, CHOL, COAD, ESCA, GBM, LIHC, LUAD, LUSC, KIRP, PRAD, STAD, THCA, READ and UCEC. A lower FBXW9 expression was only in KICH (**Figure 2G**). We further explored that the higher expression of FBXW10 in several cancers including LUAD, LUSC, LIHC, PRAD and STAD compared with normal tissues. Meanwhile, the FBXW10 expression reduced in KICH, KIRC, and THCA (**Figure 2H**). In addition, the lower FBXW11 expression level was discovered in BLCA, BRCA, GBM, LUSC, HNSC, KIRC, KIRP and UCEC. The higher expression level of FBXW11 was found in CHOL and LIHC (**Figure 2I**). We also discovered FBXW12 was more expressed in BLCA, BRCA, LIHC, LUSC, CHOL, COAD, STAD, PRAD and UCEC. In contrast, the lower FBXW12 was found in GBM, KICH, KIRC, KIRP, and THCA (**Figure 2J**). Since FBXW1/7 are the central genes of this family and have same motifs, we also analyzed transcriptional expression of these two genes in four common cancer cell lines (breast, lung, colorectal and renal cancer). The results showed FBXW1 expression in COAD was higher than matched normal cell and the expression of FBXW7 in COAD was not different from that in normal group. However, the transcription levels of the other three cancer cells were not completely consistent with the TCGA database results. A small number of cell lines selected may be one of the reasons (**sFigure 3**).

**Figure 3:**
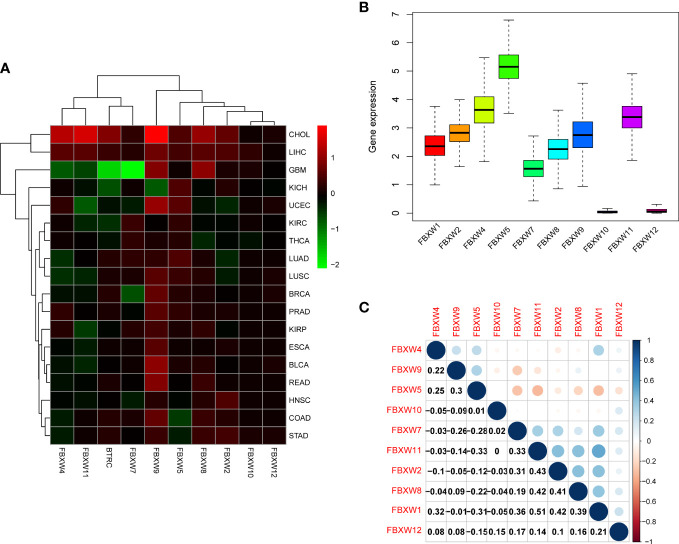
FBXW family gene expression levels and correlation in different cancer types from TCGA. **(A)** Heatmap showing the difference of FBXW family gene expression in 18 cancer types from TCGA database. The red and green indicate the high or low expression, respectively. **(B)** Boxplot illustrating the distribution of FBXW family gene expression in various cancers. **(C)** The correlation between FBXW family genes. The blue dot indicated the positive correlation. The red dot indicated the negative correlation.

We further analyzed FBXW gene family expression and correlation in various cancer types by TCGA database. The results were shown that FBXW1/4/9/11 were highest expression in CHOL (**Figure 3A**). we further investigated that FBXW5 was highly expressed, FBXW10 and FBXW12 were lowly expressed, and other members were moderately expressed in pan-cancer (**Figure 3B**). In terms of all these 18 types of cancer, two genes with most significant positive correlation were FBXW1 and FBXW11, whereas the correlation of FBXW5 and FBXW7 was completely opposite (**Figure 3C**). For the above researched cancers (BRCA, COAD, LUAD and KIRC), FBXWs as a whole showed a positive relationship, including FBXW1 and FBXW7 (**sFigure 4**) (These findings confirmed the synergistic relationships among FBXW members and revealed that the SCF complex may have a more competitive and complicated mechanism than we thought.

### The association between clinical characteristics, tumor subtypes and FBXWs expression

**Figure 4:**
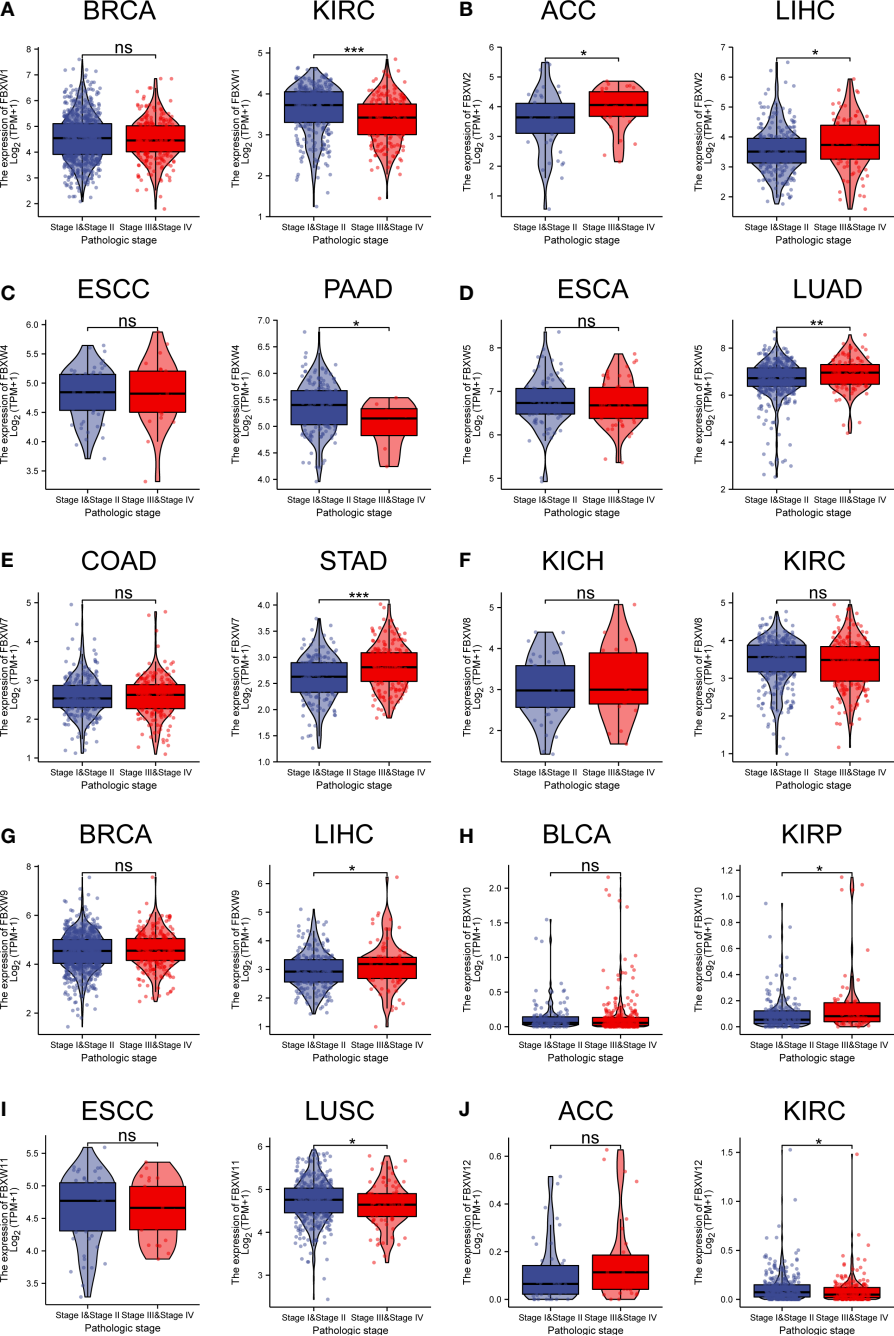
Association of FBXW family **(A–J)** gene expression with the clinical stages for different cancer types. ∗P< 0.05, ∗∗P< 0.01, and ∗∗∗P< 0.001, ns, No statistical significant.

**Figure 5:**
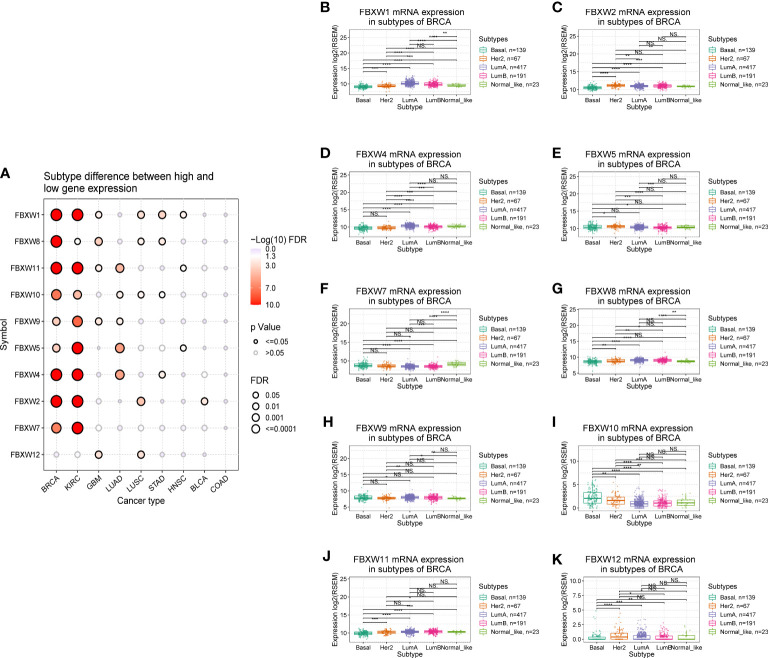
Correlation analysis between FBXW family gene expression and the subtypes of various tumors. **(A)** Subtype differences between high and low gene expression of FBXWs. **(B–K)** the relationship between FBXWs expression and the subtype of BRCA. ∗P< 0.05, ∗∗P< 0.01, ∗∗∗P< 0.001 and ****P<0.0001.

In the examination on the tumor stage relevance, we discovered that some FBXW genes expression significantly increased in tumor early stage, such as FBXW1/12 in KIRC, FBXW4 in pancreatic adenocarcinoma (PAAD), and FBXW11 in LUSC. Whereas, other genes expression predominantly increased in tumor advanced stage, including FBXW2 in ACC, FBXW2/9 in ILHC, FBXW5 in LUAD, FBXW7 in STAD, and FBXW10 in KIRP/Esophageal Squamous Cell Carcinoma (ESCC) (**Figure 4**).Moreover, we further explored the relationship between FBXWs expression levels and tumor grade. The result was indicated that FBXW4/7/11 expressions were significantly increased in KIRC early grade. In contrast, the lower expressions of FBXW1/8/9 were found in LIHC early grade, And the lower FBXW2/5/11 expressions were analyzed in the early grade of HNSC (**sFigure 5**). In addition, we analyzed the FBXWs expression levels in different subtypes of nine cancers. As shown in **Figure 5A**, the protein expression levels of most FBXWs were obviously different in BRCA and KIRC. Furthermore, we found that the expression of FBXW1/7 were significantly different in four subtypes of BRCA containing Basal, Her2, LumA, LumB compared with normal_-_like subtype. However, there were no notable difference of FBXW2/4/5/10/11/12 expression levels between LumA/LumB and Normal_-_like subtype (**Figures 5B–K**).

### Prognostic value of FBXWs in pan-cancer

**Figure 6:**
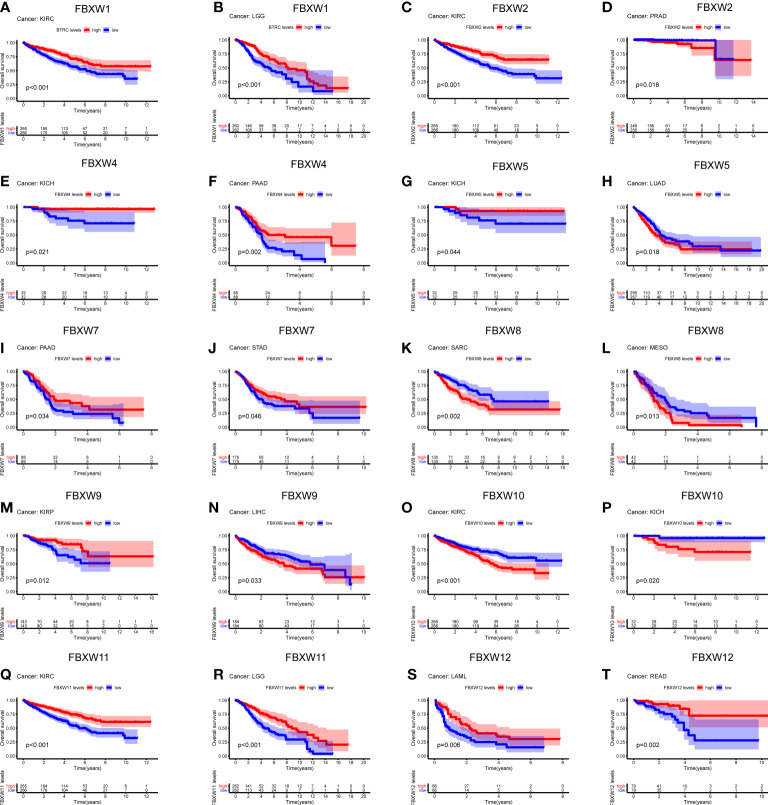
Survival analysis of FBXW family genes **(A–T)** across multiple cancer types. The red line in the photos indicates high expression and the blue line in the photos indicates low expression. P value less than 0.05 is considered as difference.

Aiming to investigate the relationship between FBXWs expression level and prognosis value, significant survival analysis with Kaplan-Meier survival curves was performed in the research (**Figure 6**; **sFigure 6**). FBXW1 was a beneficial factor in LGG, KIRC, MESO, and UVM. Besides, FBXW5 was a protective factor in KICH and UCEC. Nevertheless, FBXW5 was a high-risk factor in LUAD and ACC. The results also shown that among patients with PAAD, STAD, HNSC and SKCM, high FBXW7 expression was related with longer survival times. FBXW9 played a protective role in three different cancers, which contained KIRP, DLBC and HNSC. While, FBXW9 had a detrimental role in other three cancers including LIHC, ACC, and LGG. FBXW12 was a protective prognostic gene in LAML, READ, and UVM. FBXW12 was a high-risk gene in THYM. In addition, we found that FBXW1/2/11 played a protective role in KIRC. In contrast, FBXW10 was a dangerous factor in KIRC. On the one hand, FBXW1/11 were low-risk factors in LGG patients. On the other hand, high expressions of FBXW2/9 were associated with worse in LGG.

**Figure 7:**
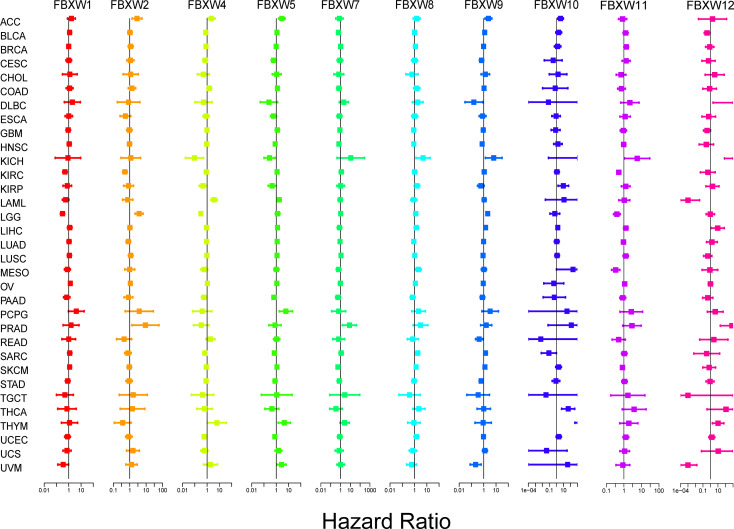
Correlation analysis of FBXW family gene expression with survival by the COX method in different types of cancers. Different colored lines indicate the risk value of different genes in tumors, hazard ratio <1 represents low risk and hazard ratio >1 represents high risk.

Then, we explored the prognosis risk of FBXWs via COX analysis. As shown in **Figure 7**, FBXW1 was a protective gene in LGG. FBXW2 had protective roles in KIRC. However, FBXW2 was high-risk factor in ACC, LGG, and PRAD. FBXW4 was a low-risk factor in KICH and LGG. For another, FBXW4 was a detrimental factor in LAML. FBXW5 acted as a detrimental factor in ACC, PCPG, and THYM. FBXW7 played a detrimental role in PRAD. FBXW8 functioned as a high-risk gene in KICH. FBXW9 had a protective effect in UVM. In contrast, FBXW9 acted as a high-risk factor in KICH and LGG. Among patients with KIRP, THCA, and THYM, high FBXW10 expression was associated with worse prognosis. While in patients with KIRC, LGG, and MESO, those with high FBXW11 expression had better prognosis. Our results also indicated that FBXW12 played a protective role in LAML and UVM. Contrarily, FBXW12 was a high-risk prognosis factor in DLBC, KICH, and PRAD (**Figure 7**). These results indicated that FBXWs in different cancers may play a different role in prognosis value.

### Genetic alterations of FBXW gene family in pan-cancer

**Figure 8:**
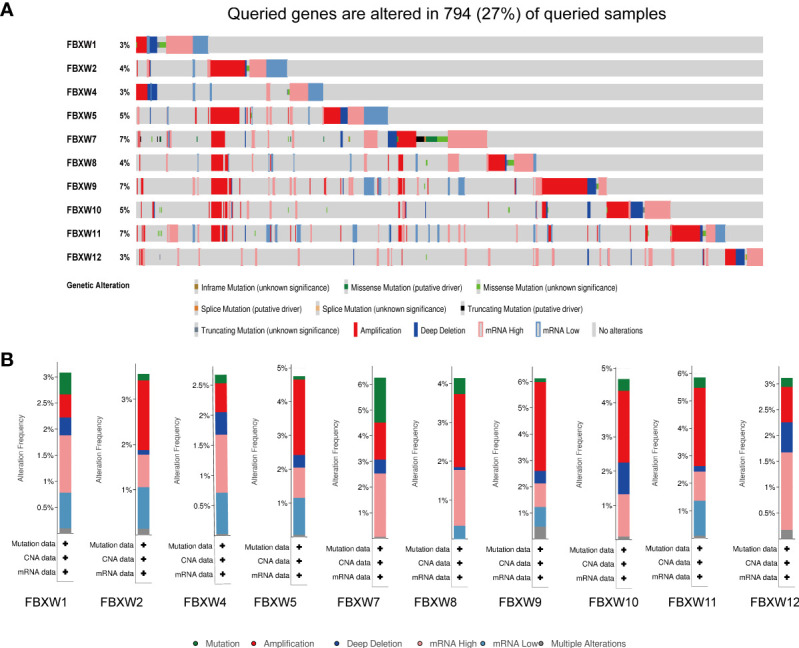
Genetic alterations and correlation analysis of FBXW family members in Pan-cancer. **(A)** summary of alteration rates for FBXW family from TCGA database using cBioPortal. **(B)** Genetic alteration frequency data of FBXW family members in pan-cancer, respectively.

We further analyzed the variation frequency and types of the FBXW gene family in 2922 cancer patients via cBioPortal database. The results indicated that gene change rates of FBXW7/9/11 were approximately 7%, which was highest in the FBXW family. Nevertheless, FBXW1/4/12 had lowest variation rates, which was only 3% (**Figure 8A**). Besides, we found that the main genetic alteration types containing amplification, mRNA high expression, mutation and deep deletion. The FBXW2/5/8/9/10/11 possessed the highest amplification rates. Meanwhile, the change type of FBXW1/4/7/12 were mainly mRNA high expression (**Figure 8B**). In conclusion, FBXW family members were likely to mutate in various cancer types.

### Relationship between FBXWs expression and tumor microenvironment in pan-cancer

**Figure 9:**
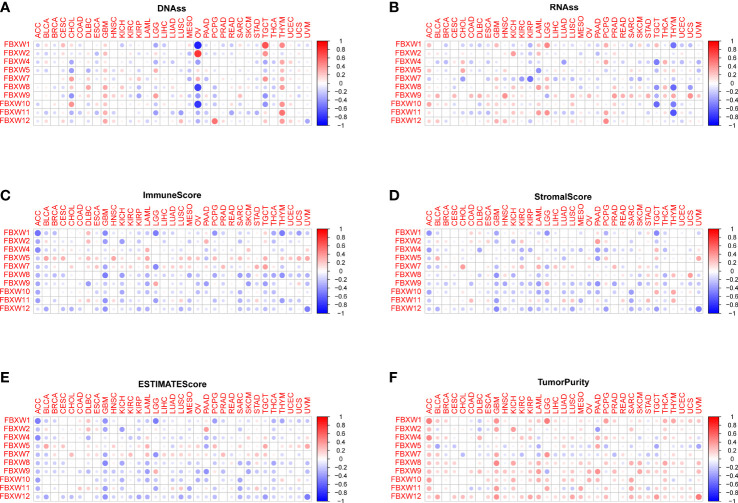
Association between FBXW family gene expression and tumor micro-environment factors and stemness score in pan-cancer. The FBXW family gene associated immune score **(A)**, stromal score **(B)**, estimate score **(C)**, DNAss **(D)**, RNAss **(E)** and tumor purity **(F)** are illustrated.

Cancer stem cell played a key role in tumor proliferation, migration, metastasis, epithelial-to-mesenchymal recurrence, and therapy resistance (74). Therefore, we explored the association between FBXWs expression and stemness score. As we can see from **Figure 9**, the expression of FBXW family had a positive or negative relationship with DNAss and RNAss in pan-cancer. For example, FBXW1/8/10 expression were negatively related with DNAss in OV. At the same time, we found that FBXW2 was positively associated with DNAss in OV. Besides, FBXW1 had a significantly positive relation with DNAss in TCGA and THYM (**Figure 9A**). During the analysis of RNAss, we observed that FBXW1/8/10/11 were negatively associated with RNAss in THYM. FBXW7 was negatively related with RNAss in several cancers such as CHOL, KIRC, and KIRP (**Figure 9B**). Furthermore, the tumor microenvironment related score results indicated most of FBXWs expression was significantly negatively associated with stromal score, immune score, and estimate score and positively related to tumor purity (**Figures 9C–F**). Specifically, we could find that FBXW5 appeared to have a consistent positive relationship across all cancers in these scores; on the other hand, FBXW12 had a consistent negative relationship. Moreover, we also discovered there was an obvious relation between immune score and FBXWs in ACC, GBM and LAML. These results demonstrated that FBXW members vary in their ability to regulate the immune microenvironment in different cancers.

### Correlation between immune infiltration and FBXWs expression

**Figure 10:**
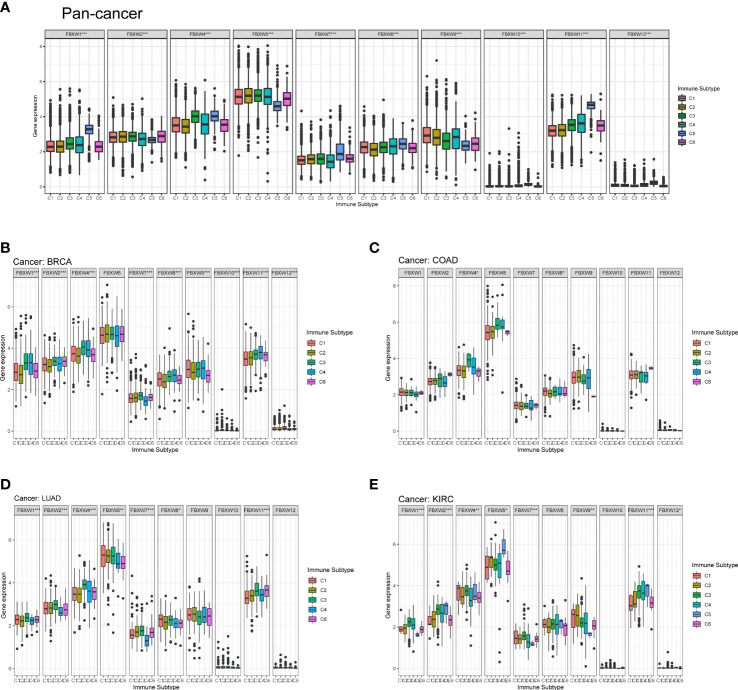
FBXW family gene expression level of different immune subtype in pan-cancer, and specific four cancer types. **(A)** In pan-cancer. **(B)** In BRCA. **(C)** In COAD. **(D)** In LUAD. **(E)** In KIRC. X axis represents immune subtype, Y axis represents gene expression. * *P<*0.05; ** *P<*0.01; *** *P<*0.001. C1: wound healing, C2: IFN-gamma dominant, C3: inflammatory, C4: lymphocyte depleted, C5: immunological quiet, and C6: TGF-beta dominant.

Then, we analyzed the relation between FBXWs and immune subtype. As was shown in **Figure 10**, The expression of all FBXW gene family members were significantly associated with immune subtype C1(wound healing), C2 (IFN-gamma dominant), C3

(inflammatory), C4 (lymphocyte depleted), C5 (immunological quiet), and C6 (TGF-beta dominant) in pan-cancer (**Figure 10A**). Then, we selected four cancer types to analyze including BRCA, COAD, LUAD, and KIRC. FBXW1 was higher expressed in C3 and C4, the expression of FBXW7 up-regulated in C2 and C3 (**Figure 10B**). FBXW4 and FBXW8 were significantly related to the immune subtype in COAD (**Figure 10C**). Furthermore, we found that FBXW1/2/4/5/7/8/11 were significantly different in LUAD, and these genes overexpressed in C3 (**Figure 10D**). The **Figure 10E** indicated that the expressions of FBXW1/2/4/5/7/9/11/12 were associated with immune subtype in KIRC. FBXW1/2/4/7 were higher expressed in C3. Meanwhile, the expression of FBXW5 was higher in C5 (**Figure 10E**). However, only FBXW5 and FBXW9 on the relation of immune subtypes have statistical significance in ACC, and FBXW11/12 was the same in GBM (**sFigure 7**). These results once again demonstrated the specificity of FBXW members in various tumors.

**Figure 11:**
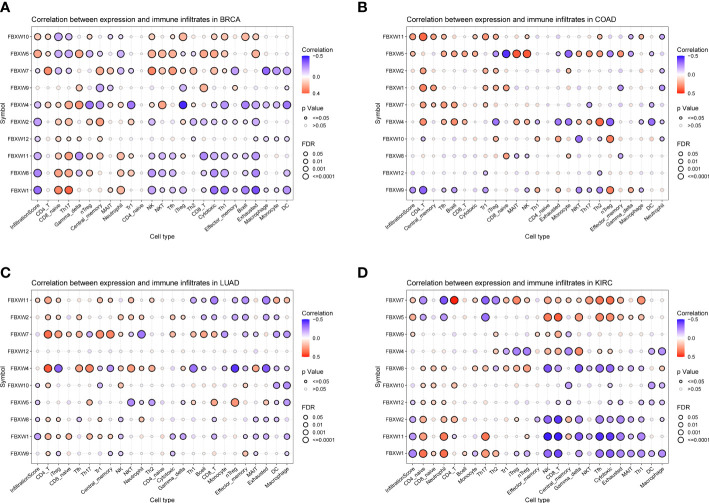
Association analysis of FBXW family gene expression with the immune-infiltration cells in four types of cancer. **(A)** in BRCA. **(B)** in COAD. **(C)** in LUAD. **(D)** in KIRC.

**Figure 12:**
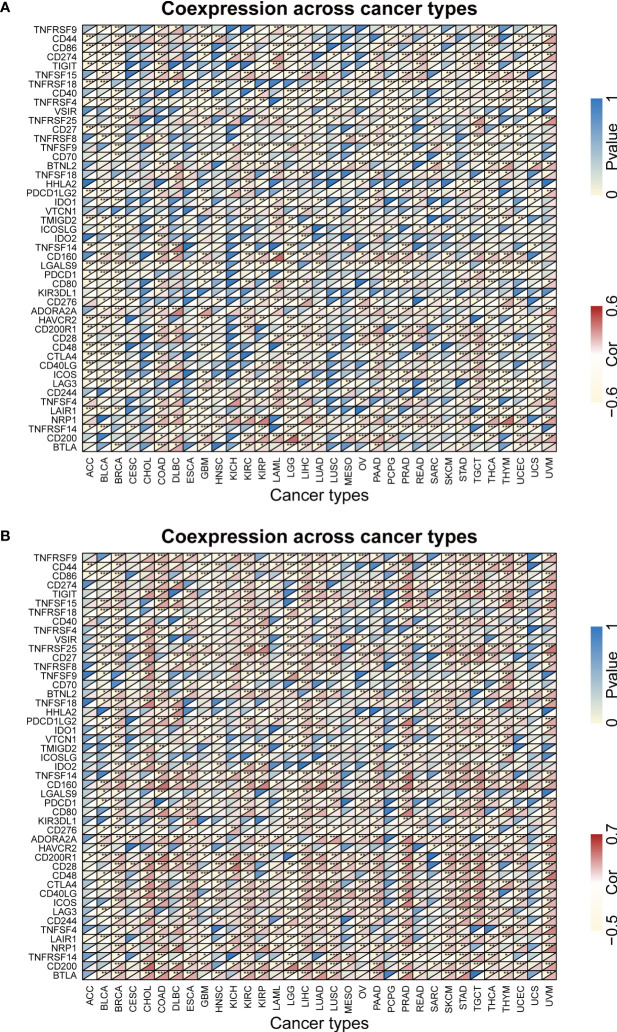
The relationship between FBXW1/FBXW7 and immune checkpoints. **(A)** Heatmap illustrating the relationship between FBXW1 and known immune checkpoints. **(B)** Heatmap illustrating the relationship between FBXW7 and known immune checkpoints. ∗P< 0.05, ∗∗P< 0.01, and ∗∗∗P< 0.001.

Previous study had demonstrated that FBXW1/7 were involved in the immune process in different ways (75–77). To further verify their immune role on the influence of tumor development and outcome, the association between immune cell infiltration levels and FBXWs expression was also evaluated. As indicated in **Figure 11**, most of FBXWs expression had significantly positive or negative relationship in these cancers, especially in BRCA and KIRC. FBXW1 expression was significantly negative association with immune-active cells such as NK, CD8_-_T, cytotoxic, and Th1, etc. However, FBXW7 as tumor suppressor factor had a positive correlation with immune-active cells and negative association with immune-suppressive cells such as Exhausted T cells, Th17, and macrophage (**Figures 11A–D**). Based on the above TME analysis results, we also investigated the correlation between FBXWs and infiltration score in ACC, GBM and LAML and found that it was not obvious (**sFigure 8**) In order to validate the results, we further investigate the relationship between FBXW1/7 expression and immune cells in pan-cancer. FBXW1 as an oncogene and FBXW7 as a tumor suppressive gene also show opposite relationships on immune infiltration cells (**s.Figure 9**). In addition, the TCGA database was used to conduct co-expression analysis, which can reveal the association between FBXW1/7 expression and immune checkpoints in pan-cancer. As shown in **Figure 12A**, FBXW1 was negatively correlated with these representative immune markers in ACC, BLCA, BRCA, PCPG, UCEC and UCS. However, FBXW7 was positively associated with these immune checkpoints in most cancers (**Figure 12B**). The opposite relationship between FBXW1 and FBXW7 in immune infiltration may be the reason for the different prognosis of cancer patients.

### Drug sensitivity analysis of FBXW gene family

Drug resistance has always been an insurmountable problem in clinic treatment, and also a prominent factor in the development of adverse outcomes for many patients. Previous study reported that FBXWs has been validated to involve in drug resistance development through proteolytic regulation of substrates (20). For example, abnormal expression of FBXW7 could increase doxorubicin resistance in COAD, and in tamoxifen-resistant breast cells, FBXW1 expression was significantly upregulated.

Aiming to further explore the role of FBXWs in chemotherapy or targeted therapy, the integrated analysis between drug sensitivity and FBXW gene expression data was also performed in this study. The result from CellMiner™ was shown that FBXW7 expression had a positive relationship with drug sensitivity of Nelarabine and Chelerythrine. In addition, FBXW11 expression had a significantly negative relation with the drug sensitivity of Imatinib, LDK-378, and Palbociclib. FBXW9 expression was negatively associated with Dasatinib’s drug sensitivity and positively related to drug sensitivity of tfdu (**sFigure 10**). These results may bring some implications for clinical medication based on FBXWs drug sensitivity.

## Discussion

In consideration of the vital roles of FBXW gene family in progression of numerous cancers, it is of great importance to study the expression patterns and prognostic effect of FBXW gene family in multiple cancers, which could facilitate tumor early diagnosis (78). In our study, we firstly performed gene structure and motif analysis of the FBXWs, and found that they have obvious individual differences which may lead to functional differences in FBXW genes. Then, we also summarized previous reports on the function of the FBXW family in multiple cancers, and found that FBXW1/5 act as oncogenes in many cancers, however, FBXW2/7 exert the antitumor properties (**Table 1**). Specifically, from the perspective of the transcription level, we found that FBXW1 was significantly up-regulated in six different cancers, which was consistent with most previous studies (79). Whereas the abnormal expression of FBXW2 was related to twelve different cancers, seven of which were upregulated and five were down-regulated. FBXW5/9/12 were overexpressed in most cancers, however, FBXW11 expression in pan-cancer was opposite. Notedly, FBXW7, as a tumor suppressor, was highly expressed in six cancers (CHOL, KIRC, KIRP, LIHC, LUAD and THCA) which may be one of the reasons affecting the outcome of these cancers. Besides, we analyzed the FBXWs expression in different tumor stages and grades. The results indicated the expression of most genes was significantly different in the development of various cancers, which inspired us to consider the roles of FBXW family genes as diagnosis and prognosis markers in pan-cancer.

We also summarized all meaningful survival-related results and found that FBXW members accounted for the most significant proportion in KIRC among the above mentioned four common cancers (BRCA, COAD, LUAD and KIRC) (**Supplementary Table 1**).Additionally, we also demonstrated that FBXW1 was a protective factor in LGG, which was consistent in different database. The overexpression of FBXW2 was related to worse OS in ACC, LGG, and PRAD. However, Zhou et al.’s study indicated that FBXW2 could be a tumor suppressor of PRAD by promoting EGFR ubiquitination and degradation (41), which seems to be contrary to our results. The reason for this phenomenon may be that FBXW2 itself is prone to mutation which could change the overall prognosis tendency (37), or it may be that FBXW2 needs to be combined with other ligand proteins to form SCF complexes, which determine carcinogenicity depending on the kind of ubiquitinated substrates, thereby playing itself function and maintaining itself activity (80–82). Therefore, artificial obvious changes in the expression level of FBXW2 in the tumor microenvironment may lead to different prognosis compared with the database analysis. Additionally, High expression of FBXW7 was significantly associated with longer survival in PAAD, STAD, HNSC, SKCM. Previous studies indicated that low expression of FBXW7 was a major cause of carcinogenesis and poor prognosis in Gastric cancer (51, 83), which was consistent with our findings. Up-regulation of FBXW11 expression was related to better prognosis in KIRC, LGG, and MESO. In short, these results suggested that FBXWs could be great prognosis-related markers in pan-cancer.

An increasing number of studies have showed that TME could exert great influence on tumor proliferation, metastasis, micro angiogenesis, and even immune escape (84–86). However, to date, the role of FBXWs in TME has not been elucidated. Our results indicated FBXW7 expression had a positive relationship with the levels of stromal score, immune score and estimate score in multiple tumors, suggesting FBXW7 is expected to efficient immunomodulatory factor. Furthermore, the correlation between immune subtypes and FBXWs expression was also examined in this study and found that most of FBXWs have a close relation with BRCA, LUAD and KIRC, however, in other cancers such as ACC and GBM, the correlations was weakened obviously. Similarly, the relation between FBXWs and infiltration score of immune cells in BRCA and KIRC was the most significant, suggesting FBXW family may be potential orientation in the immune-therapy of the mentioned four cancers. Then, we selected two most representative members FBXW1/7 for further immune infiltration analysis, and the reverse results on immune checkpoints and cells showed that they may induce diverse immune response and thereby cause markedly different endpoint of tumor progression.

In conclusion, our research unveiled complicated and comprehensive roles of FBXW family members expression in cancer progression and clinical outcome that warrant further investigation, suggesting FBXWs could be promising prognostic biomarkers in special tumors. More importantly, we identified the relationship between immune infiltration and individual members of the FBXW family in different cancers, thus providing a few direction for immunotherapy. This study, however, has some limitations. Our results derives mainly from the computational analysis of genomic data, and detailed functional mechanisms of FBXWs by vivo and vitro experiments are needed to build in the future. In addition, our study does have a limitation on the connection of FBXW family with immunotherapy. The role of FBXWs in immunotherapy should be further validated in clinical trials and cell experiments.

## Data availability statement

The datasets presented in this study can be found in online repositories. The names of the repository/repositories and accession number(s) can be found in the article/**Supplementary Material**.

## Author contributions

ZY and ZYS designed the overall programming of this study and conducted comprehensive guidance. TH and XO drafted and revised the manuscript. TH and JL performed the data analysis. BS and ZDS participated in the data collection. All authors contributed to the article and approved the submitted version.
