# Supplementary material for: Pan-cancer analysis of FBXW family with potential implications in prognosis and immune infiltration
Source: Front Immunol. 2022 Dec 14;13:1084339. doi: 10.3389/fimmu.2022.1084339 (PMC9795248; doi:10.3389/fimmu.2022.1084339)
Supplement: Supplementary file 1 [file DataSheet_1.docx]

Supplementary Material

Supplementary Table 1

Summary of the results of the FBXW family survival analysis

| **FBXW members** | **Cancers with survival significance** |
| --- | --- |
| FBXW1 | KIRC, LGG, MESO, UVM |
| FBXW2 | ACC, KIRC, LGG, PRAD, THYM |
| FBXW4 | ACC, BLCA, KICH, LAML, LGG, PAAD, PCPG, THYM, UCEC |
| FBXW5 | ACC, CESC, DLBC, KICH, LAML, COAD, SARC, UCEC, UVM |
| FBXW7 | HNSC, PAAD, SKCM, STAD, THCA |
| FBXW8 | MESO, SARC, UCS |
| FBXW9 | ACC, DLBC, HNSC, KIRP, LGG, LIHC, SARC, UVM |
| FBXW10 | ACC, KICH, KIRC, LGG, PRAD, SARC |
| FBXW11 | KIRC, LGG, MESO, THCA |
| FBXW12 | LAML, READ, THYM, UVM |

**Supplementary Figures**

**Figure 1:**


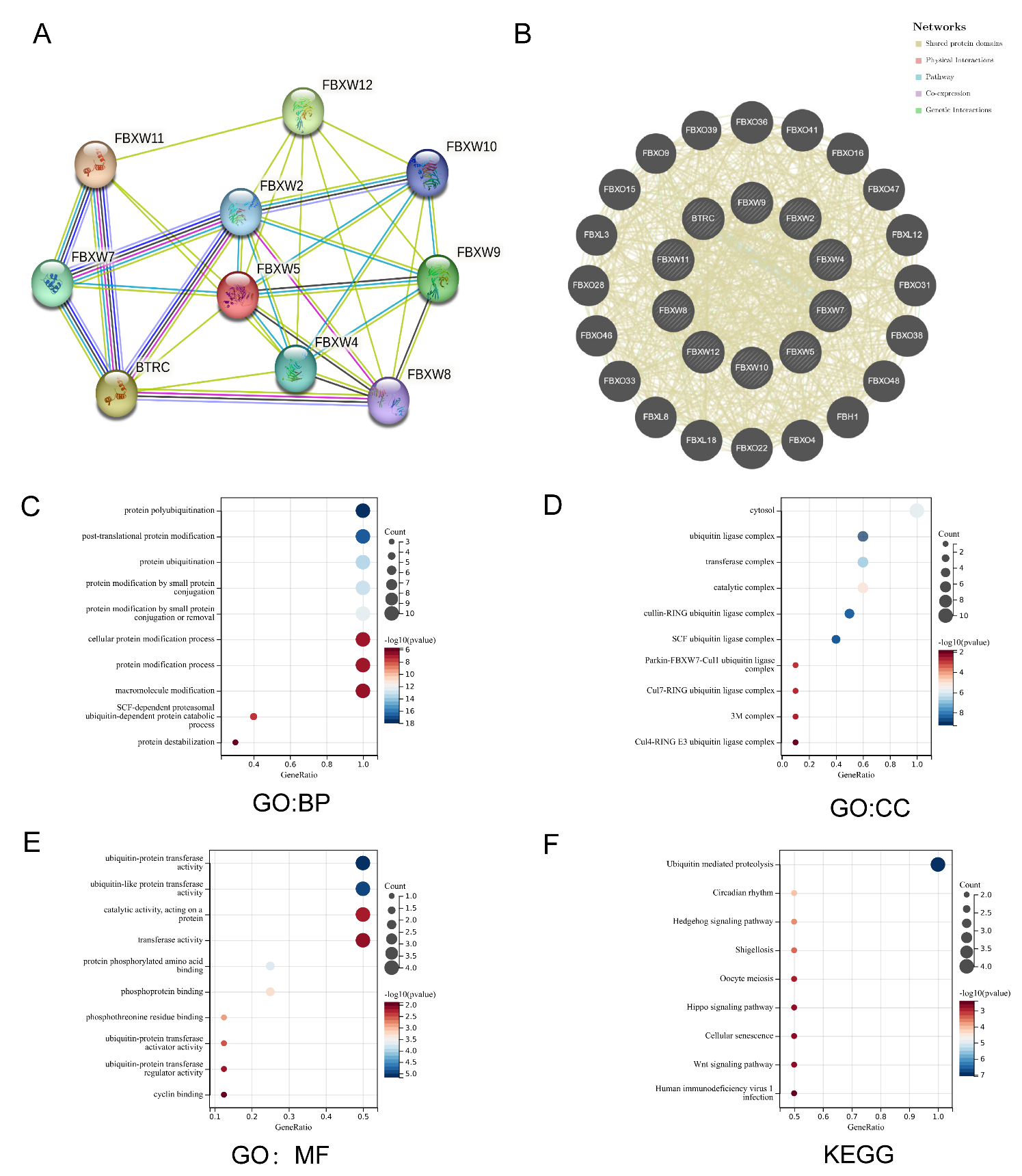


**Figure 2:**


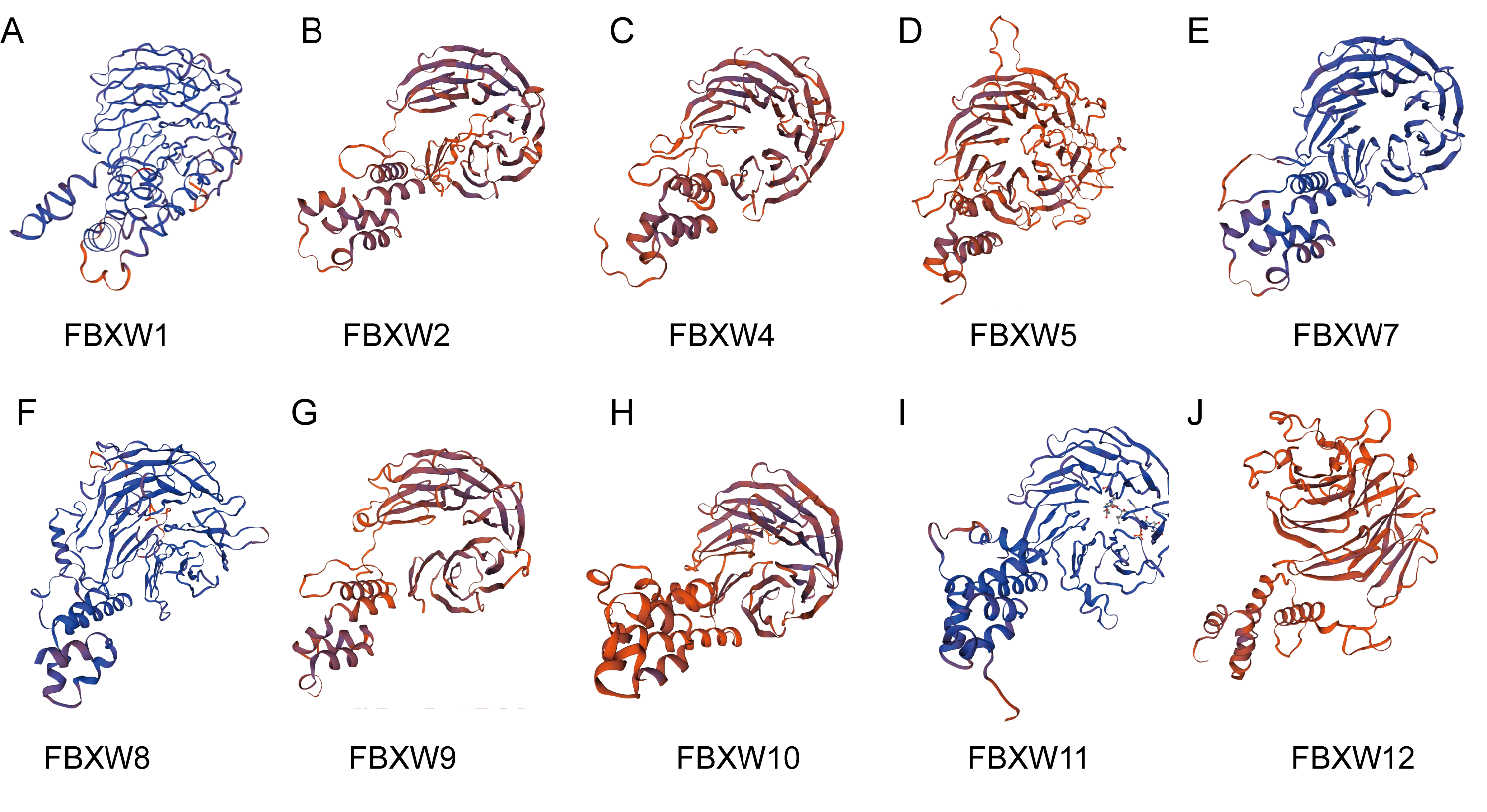


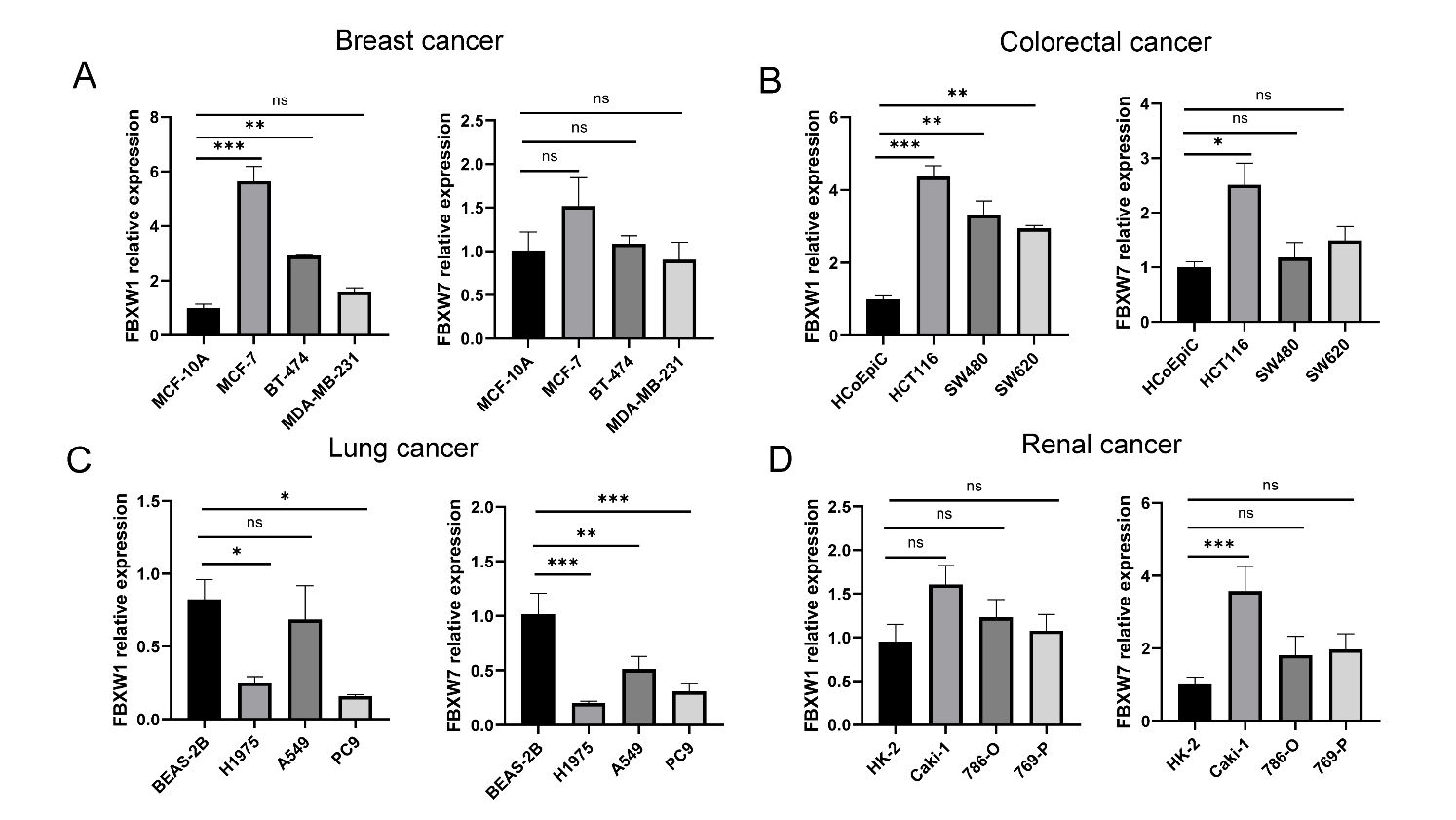
**Figure 3:**

**
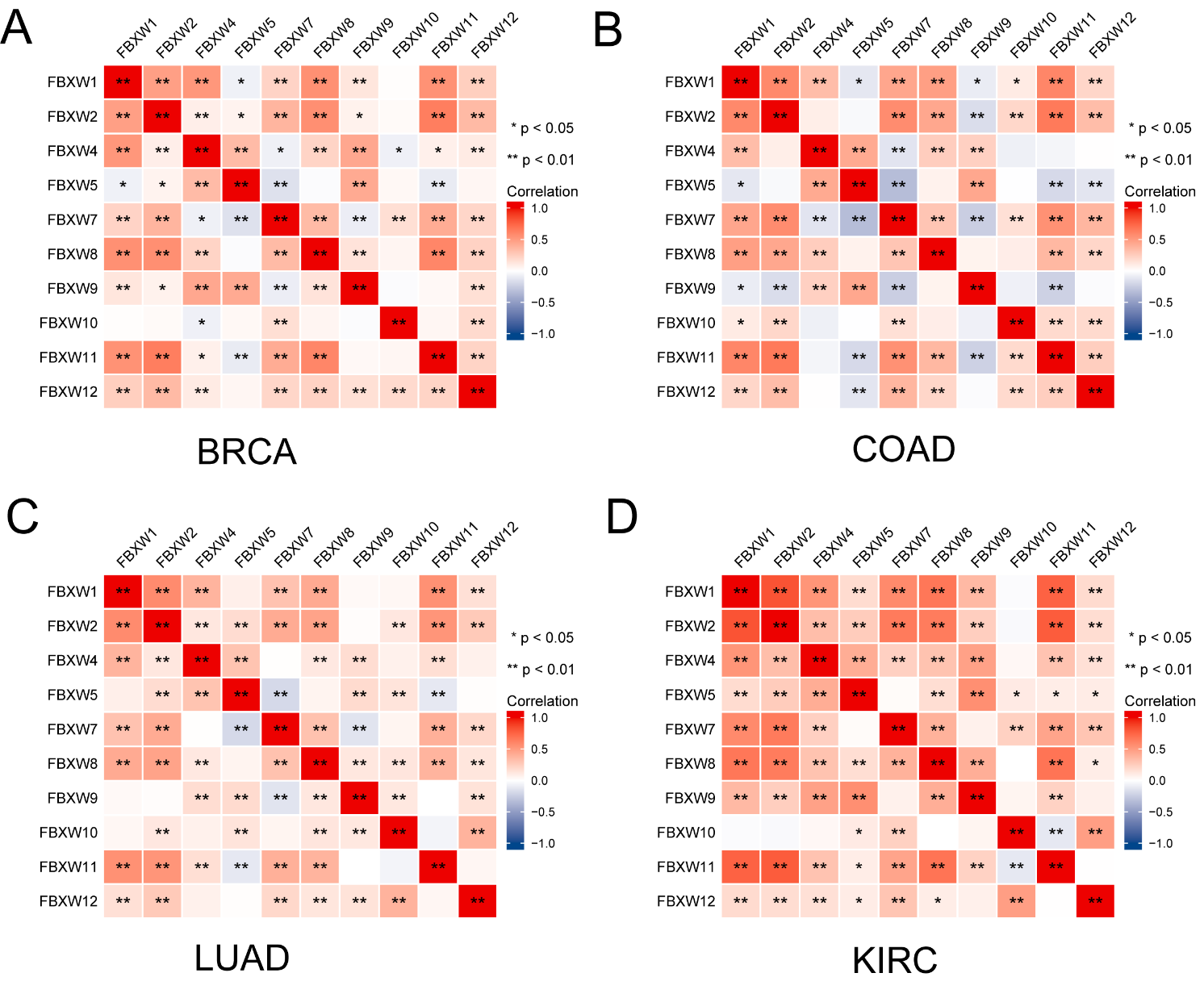
Figure 4:**


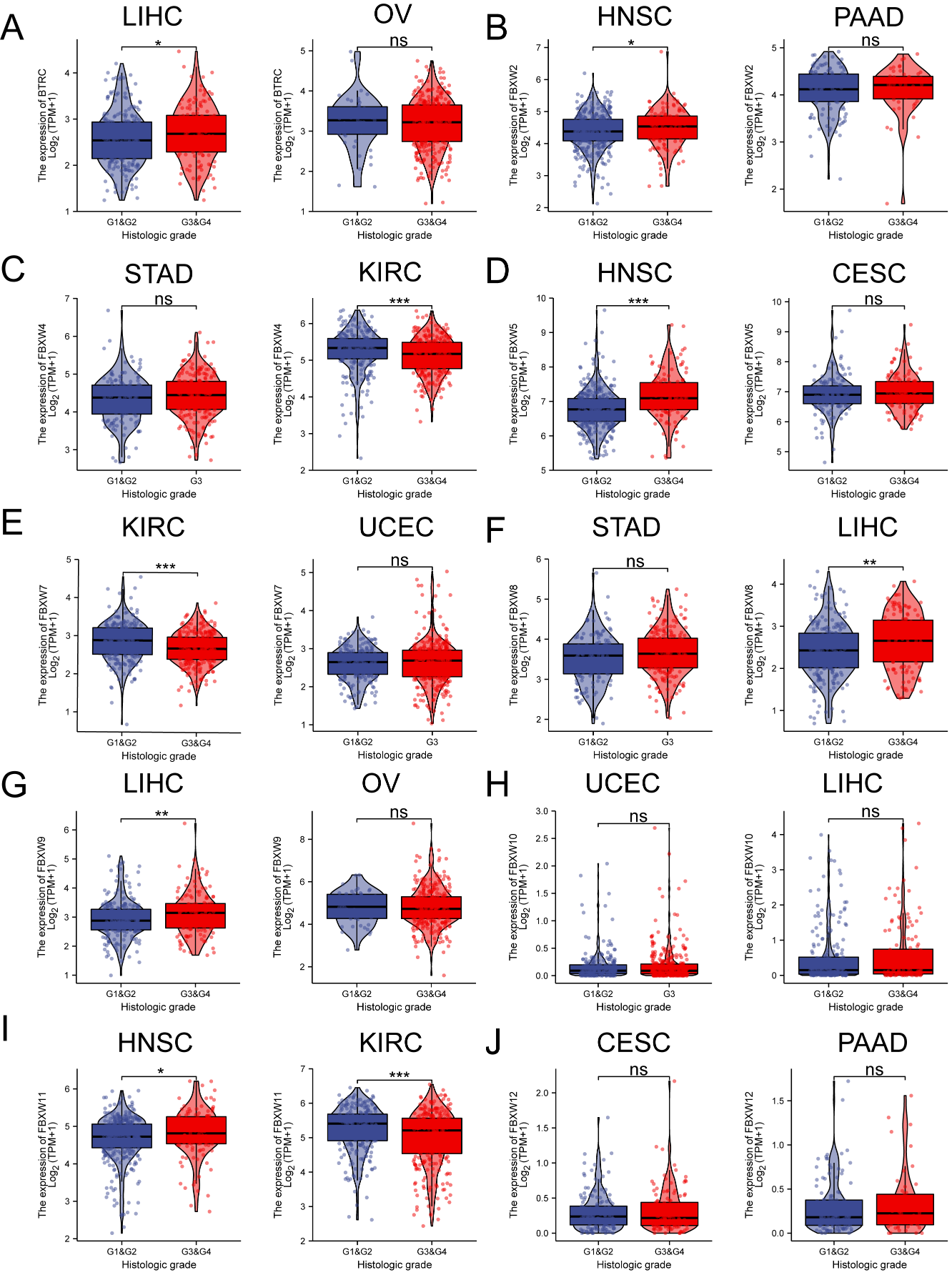
**Figure 5:**

**Figure 6:**


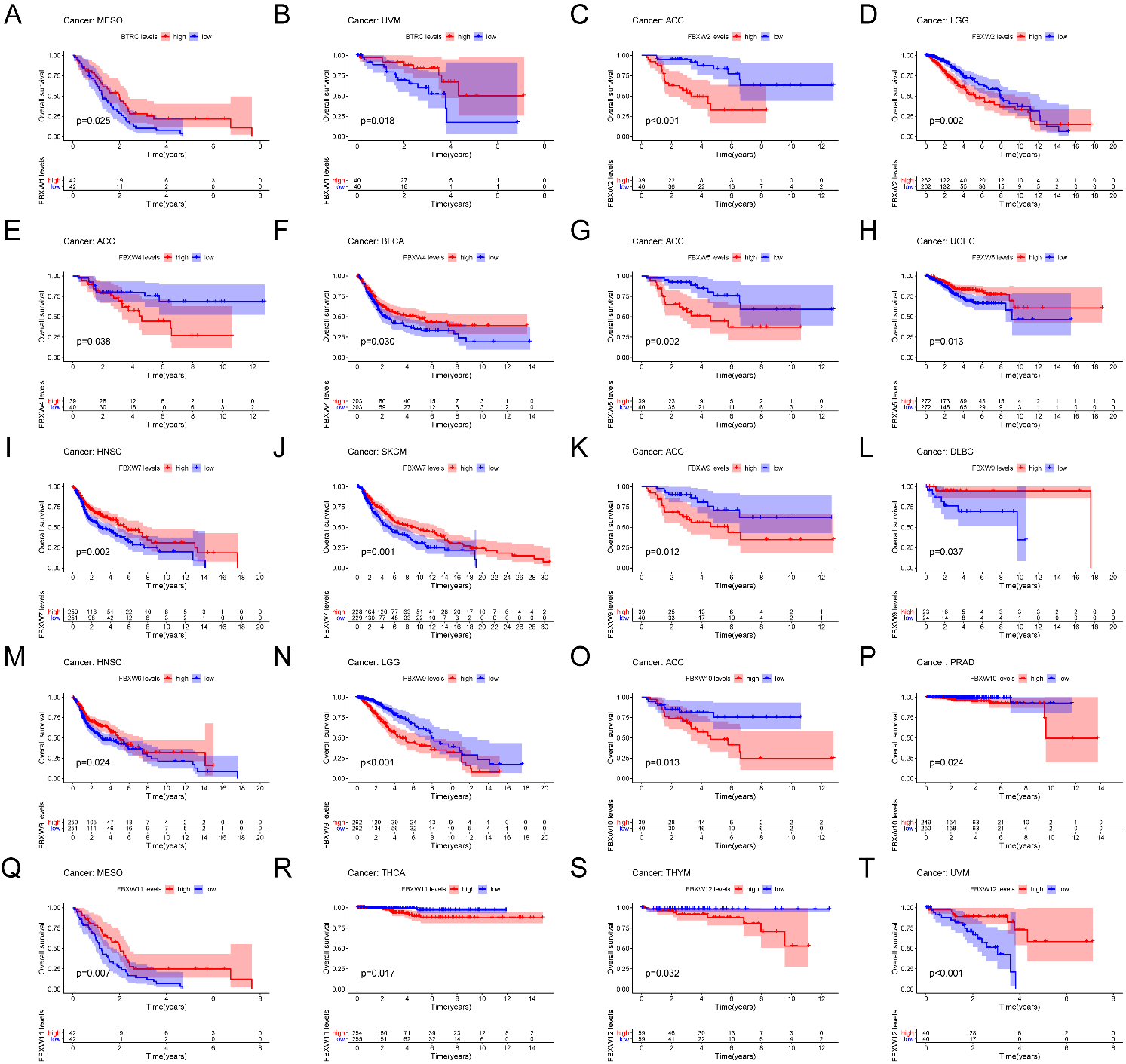


**Figure 7:**

**
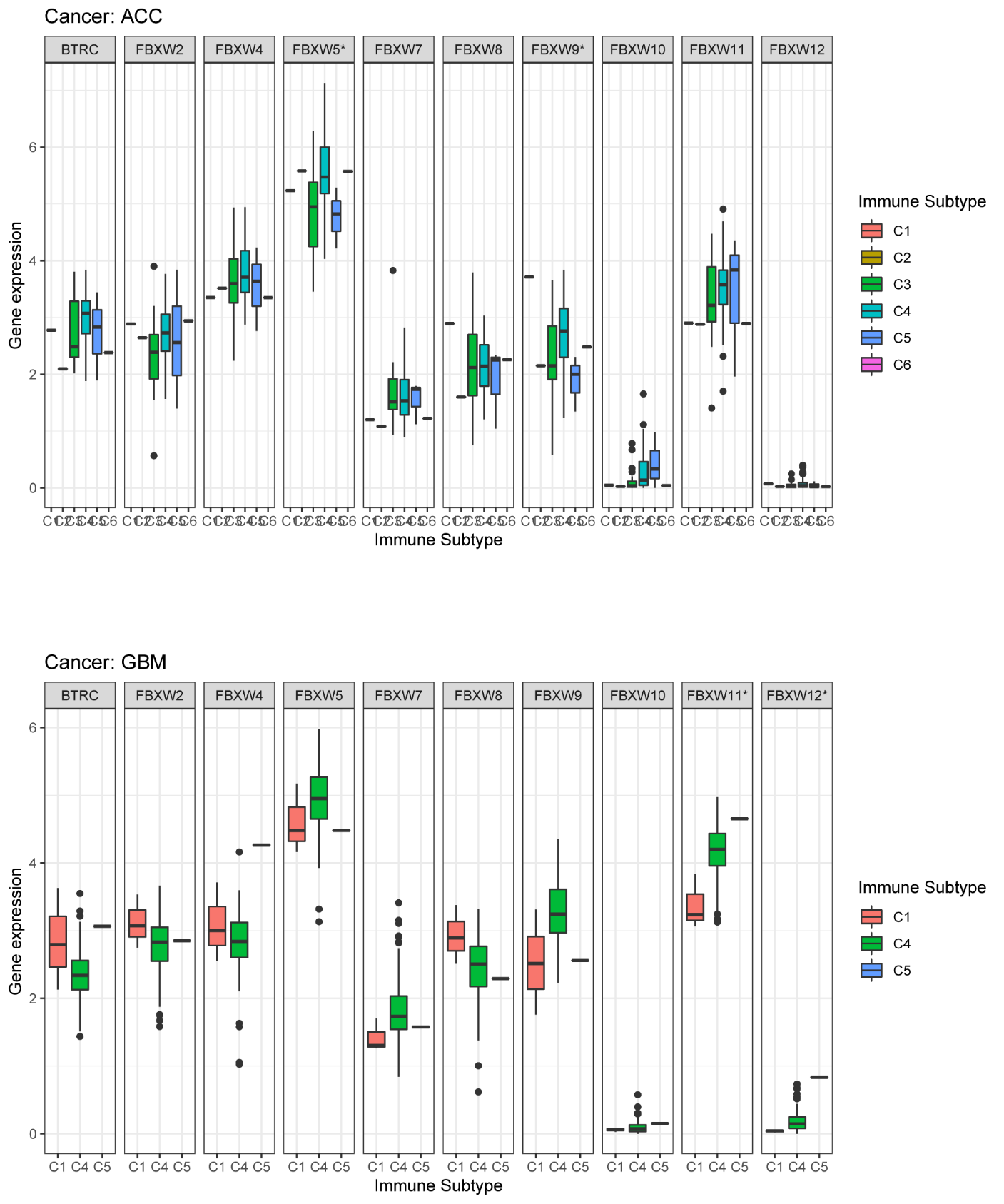
**


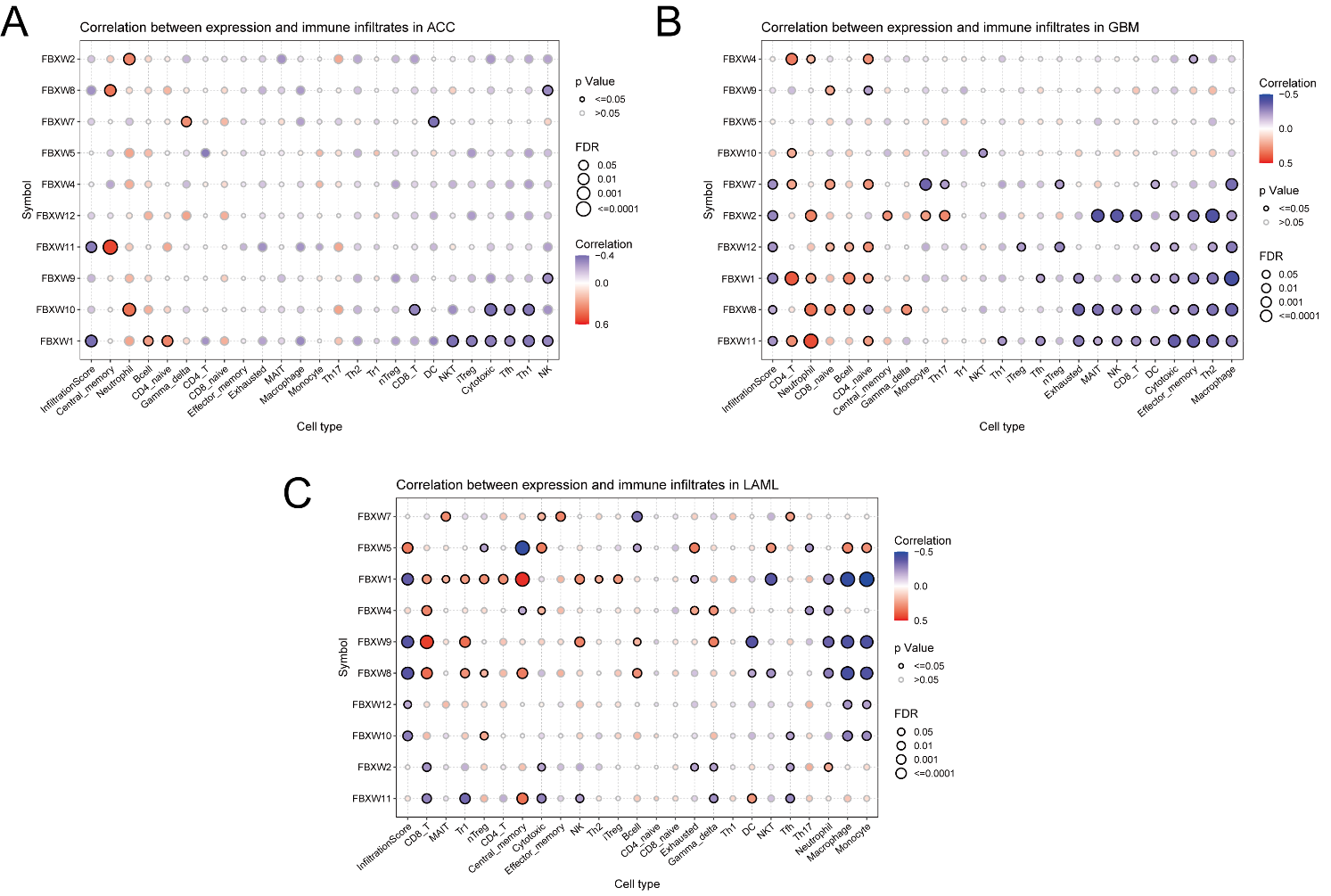
**Figure 8:**

**Figure 9:**


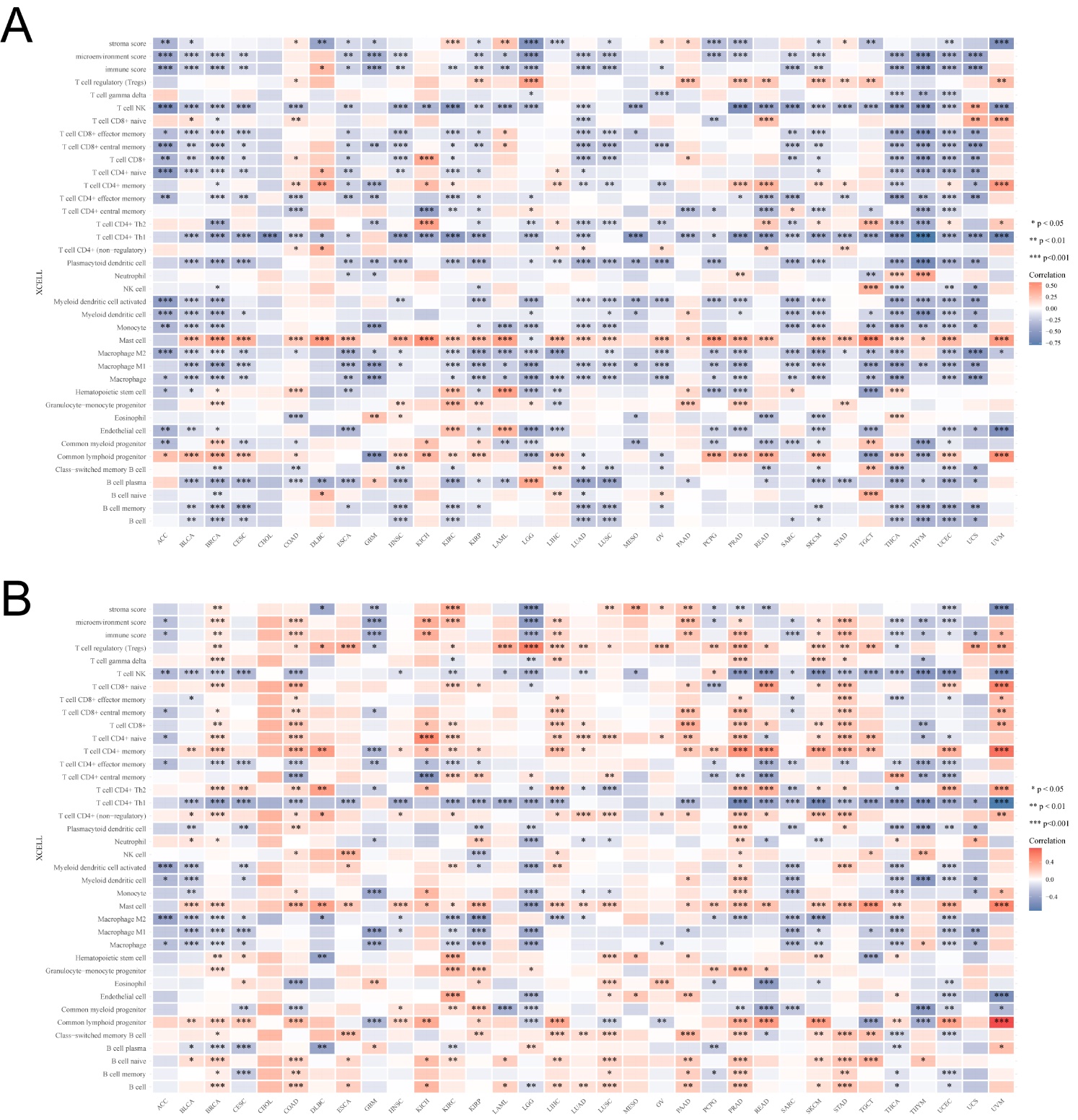


**
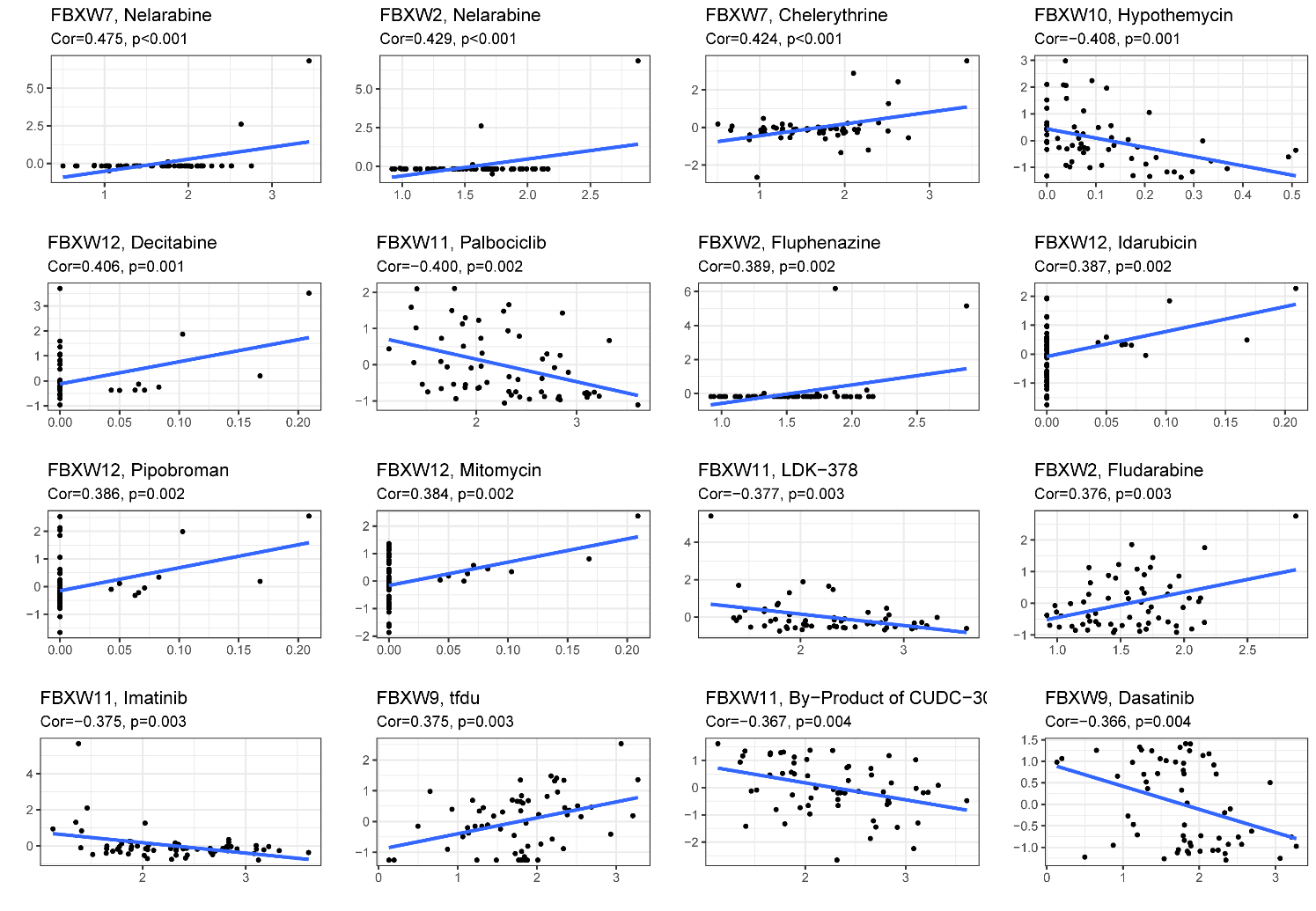
Figure 10:**

**Supplementary Figure legends**

**Figure 1.** Interaction network of the members of FBXW family. (A) PPI network construction of FBXW family members by string database. (B) PPI network construction of FBXW family members by GeneMANIA database. (C-F) The KEGG and GO enrichment pathway of FBXW family members.

KEGG: Kyoto Encyclopedia of Genes and Genomes; BP: Biological process; CC: Cell component; MF: Molecular function.

**Figure 2.** Predicted three-dimensional domains of FBXW proteins from *Homo sapiens.* 3D models of the 10 FBXWs according to SWISS-MODEL. The blue colour indicates a low-energy structure, and the orange colour indicates a high-energy structure.

**Figure 3.** Relative FBXW family members’ mRNA levels in specific four types of cancer. (A) Breast Cancer, (B) Lung Cancer, (C) Colorectal Cancer, and (D) Renal Cancer. ∗P< 0.05, ∗∗P< 0.01, and ∗∗∗P< 0.001.

**Figure 4.** The relationships between FBXW family members. (A) in BRCA. (B) in COAD. (C) in LUAD. (D) in KIRC. ∗P< 0.05, ∗∗P< 0.01.

**Figure 5.** Association of FBXW family gene expression with the clinical grades for different cancer types. ∗P< 0.05, ∗∗P< 0.01, and ∗∗∗P< 0.001.

**Figure 6.** Survival analysis of FBXW family genes across other cancer types. The red line in the photos indicates high expression and the blue line in the photos indicates low expression. P value less than 0.05 is considered as difference.

**Figure 7.** FBXW family gene expression level of different immune subtype in other two cancers. (A) ACC. (B) GBM.

**Figure 8.** Association analysis of FBXW family gene expression with the immune-infiltration cells in other three types of cancer. (A) in ACC. (B) in GBM. (C) in LAML.

**Figure 9.** The relationship between FBXW1/FBXW7 and immune infiltration cells (A) Heatmap illustrating the relationship between FBXW1 and known immune cells. (B) Heatmap illustrating the relationship between FBXW7 and known immune cells. ∗P< 0.05, ∗∗P< 0.01, and ∗∗∗P< 0.001.

**Figure 10.** Correlation analysis between FBXW family members and drug sensitivity of anticancer drugs in CellMiner. P value less than 0.05 is considered as difference.
